# RNA sequencing analysis between ruptured and un-ruptured brain AVM

**DOI:** 10.1186/s41016-022-00282-4

**Published:** 2022-06-02

**Authors:** Hao Li, Zihan Yan, Ran Huo, Xiaolong Ya, Hongyuan Xu, Zechen Liu, Yuming Jiao, Jiancong Weng, Jie Wang, Shuo Wang, Yong Cao

**Affiliations:** 1 Department of Neurosurgery, Beijing Tiantan Hospital, Capital Medical University, 119 South Fourth Ring Road West, Fengtai District, Beijing, 100071 China; 2 China National Clinical Research Center for Neurological Diseases, Beijing, China; 3 Department of Biostatistics, Harvard School of Public Health, Boston, USA; 4 Beijing Neurosurgical Institute, Capital Medical University, Beijing, China

**Keywords:** Brain arteriovenous malformation, Rupture, Inflammatory processes, Cell adhesion, Myofibril assembly

## Abstract

**Background:**

A brain arteriovenous malformation (BAVM) is a tangle of abnormal blood vessels connecting the arteries and veins in the brain and is associated with a higher risk for intracerebral hemorrhage (ICH). RNA sequencing technology has been recently used to investigate the mechanism of diseases owing to its ability to identify the gene changes on a transcriptome-wide level. This study aims to gain insights into the potential mechanism involved in BAVM rupture.

**Methods:**

Sixty-five BAVM nidus samples were collected, among which 28 were ruptured and 37 were un-ruptured. Then, next-generation RNA sequencing was performed on all of them to obtain differential expressed genes (DEGs) between the two groups. In addition, bioinformatics analysis was performed to evaluate the involved biological processes and pathways by GO and KEGG analysis. Finally, we performed a univariate Cox regression analysis to obtain the early rupture-prone DEGs.

**Results:**

A total of 951 genes were differentially expressed between the ruptured and un-ruptured BAVM groups, of which 740 genes were upregulated and 211 genes were downregulated in ruptured BAVMs. Then, bioinformatics analysis showed the biological processes and pathways related to the inflammatory processes and extracellular matrix organization were significantly enriched. Meanwhile, some downregulated genes are involved in cell adhesion and genes participating in response to muscle activity and the terms of nervous system development. Finally, one hundred twenty-five genes, many were involved in inflammation, were correlated with the early rupture of BAVMs.

**Conclusions:**

The upregulated genes in the ruptured BAVM group were involved in inflammatory processes and extracellular matrix organization. Some of the downregulated genes participated in cell adhesion and myofibril assembly, indicating the role of enhanced inflammation and reduced inflammation vessel strength in BAVMs rupture.

**Supplementary Information:**

The online version contains supplementary material available at 10.1186/s41016-022-00282-4.

## Background

Brain arteriovenous malformations (BAVMs) are a complex tangle of abnormal arteries and veins located in the brain, and this vascular abnormality lacks an intervening capillary network [1]. The risk of spontaneous intracranial hemorrhage (ICH) caused by the rupture of BAVM was estimated at 2–4% per year and can be as high as 34% under certain conditions, and the ICH could cause catastrophic neurological consequences [2, 3]. Early surgical management can effectively eliminate the cumulative lifetime risk of ICH; however, the higher rate of permanent neurological defects or death makes the treatment of un-ruptured BAVMs increasingly controversial [4]. Currently, there is no specific available medical therapy for the treatment of BAVMs. The investigation of the mechanism of BAVM rupture would shed light on developing new medical therapies, but the underlying mechanism of this process remained largely elusive.

It is likely that many cellular and molecular events played orchestrally, which eventually led to the rupture of BAVM [5]. Instead of examining individual or a few interrelated gene products, RNA sequencing technology enables the investigation of the entire transcriptome simultaneously and profiling the gene signature comprehensively. Recent RNA sequencing studies have been used to explore the pathogenesis and development of BAVM [6, 7]. However, no such studies have looked into the rupture processes of BAVM.

Thus, in this study, we classified the relatively large number of BAVM samples into the un-ruptured and ruptured groups and performed RNA sequencing analysis of these samples to unveil the underlying expression signatures along with the rupture of BAVM.

## Methods

### Patients and samples

All study participants with a defined nidus and evidence of arteriovenous shunting on digital subtraction angiography were recruited and underwent microsurgical resection in the Neurosurgical Department of Beijing Tiantan Hospital. A group of 65 BAVMs nidus was collected after the resection. Then, 31 BAVM nidus were obtained as an independent validation group. All the specimens were snap-frozen and stored in liquid nitrogen. All the participants provided written informed consent, and the institutional review board of Beijing Tiantan Hospital approved this study, Capital Medical University. BAVM patients were dichotomized as ruptured and un-ruptured groups based on the radiological examination (CT or MRI) combined with their medical history. The location of BAVM was defined as the brain lobe to which most part of the BAVM belonged.

### RNA extraction, library preparation, and sequencing

Total RNA was extracted from the BAVM nidus using TRIzol Reagent according to the manufacturer’s instructions. Then, RNA purity was determined by NanoPhotometer® spectrophotometer (IMPLEN, Germany). The concentration and integrity were examined by Qubit® RNA Assay Kit with a Qubit® 2.0 Fluorometer (Life Technologies, USA) and RNA Nano 6000 Assay Kit in a Bioanalyzer 2100 system (Agilent Technologies, USA). The detailed method, RNA sequencing data, and RNA sequencing validation using RT-qPCR could be found in our previous studies [7–9].

### Differential expressed genes (DEGs) and bioinformatics analysis

To obtain the DEGs between the ruptured and un-ruptured BAVM groups, reads containing adaptors or poly-N and low-quality reads were first removed from the raw data. Then, the clean reads were aligned with both the transcript reference and Hg19 RefSeq (RNA sequences, GRCh37) using STAR v2.2.1. The gene expression was calculated as fragments per kilobase of exon per million mapped fragment values with RSEM v1.3.0. DEGs were defined as differential expressed transcripts with *P* < 0.05 and fold change > 1.50 or < 0.67.

### GO and KEGG analysis

For further analyzing the common DEGs, Gene Ontology (GO) analysis and Kyoto Encyclopedia of Genes and Genomes (KEGG) pathway analysis were conducted on DAVID (The Database for Annotation, Visualization and Integrated Discovery, https://david.ncifcrf.gov/). An FDR value of 0.05 was set as the cutoff criterion. Gene expression data were analyzed in R v3.5.1.

### Cox regression analysis

The univariate Cox regression analysis was applied to select for BAVM early rupture-prone prognostic DEGs. DEGs associated with overall survival (OS) with *P* < 0.05 were considered statistically significant. The univariate analysis was performed using the R packages “survival” and “surveminer” to identify OS-related DEGs.

## Results

**Fig. 1:**
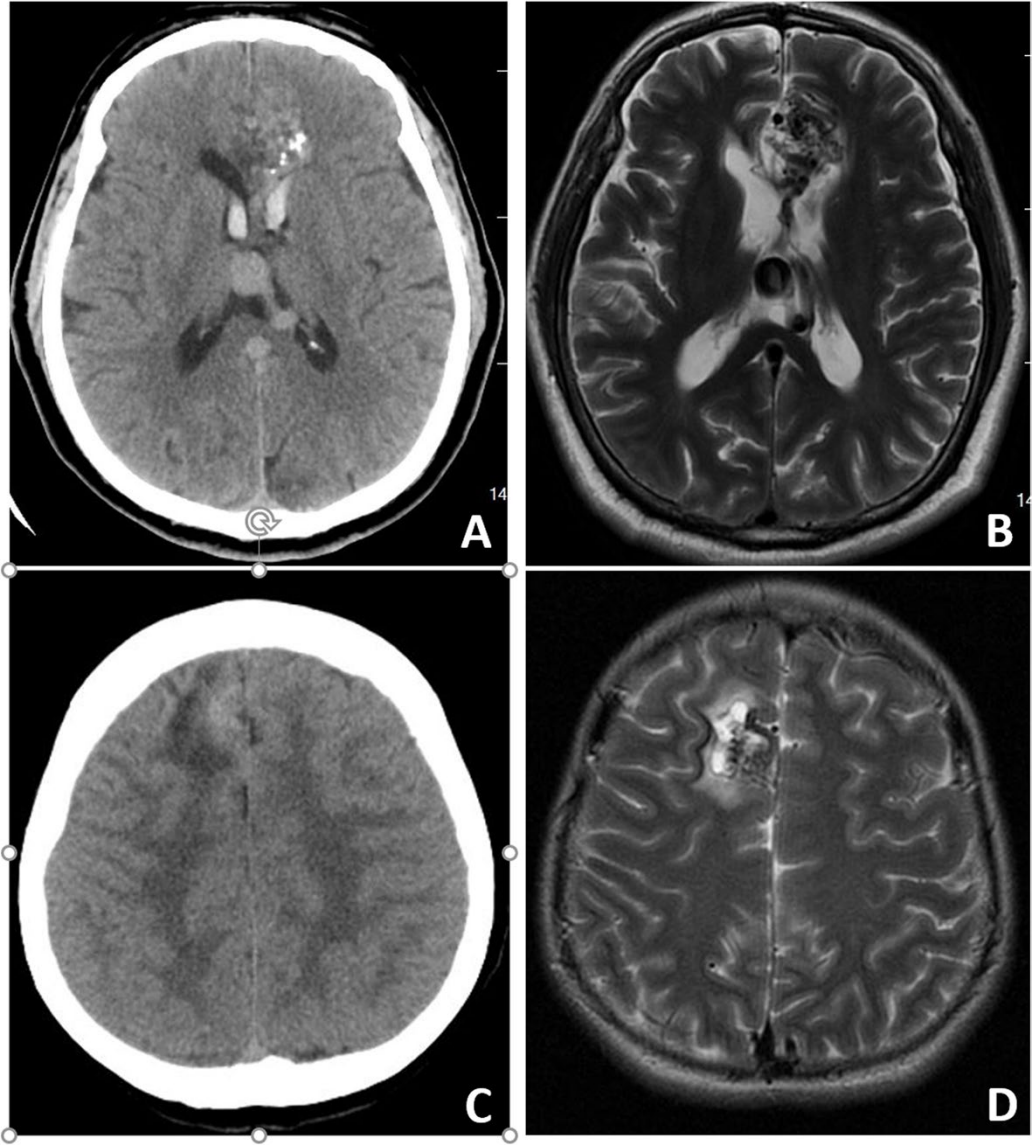
The CT (Additional file 7: Fig. S1A) and MRI (Additional file 7: Fig. S1B) of ruptured BAVM and CT (Additional file 7: Fig. S1C) and MRI (Additional file 7: Fig. S1D) of un-ruptured BAVM

The baseline clinical characteristics of the 65 BAVM patients were summarized in Table 1, among which 28 were ruptured BAVMs and 37 were un-ruptured BAVMs. The typical CT\MRI images of un-ruptured and ruptured BAVM were present in Fig. 1.

**Table 1:** Baseline characteristics of the BAVM patients

Characteristic	
Sex, female	26 (40%)
Age, years	29 ± 15 (range 2–68)
**Clinical presentation**	
Ruptured BAVMs	28 (43%)
Un-ruptured BAVMs	37 (67%)
**Main location**	
Frontal	27 (42%)
Temporal	23 (35%)
Parietal	10 (15%)
Occipital	3 (5%)
Cerebellar	2 (3%)

All of these patients were of Chinese Han ethnicity and had no BAVM family history. There is no significant difference in baseline characteristics between these two groups (Additional file 1: Table S1).

### Differential expressed genes analysis

**Fig. 2:**
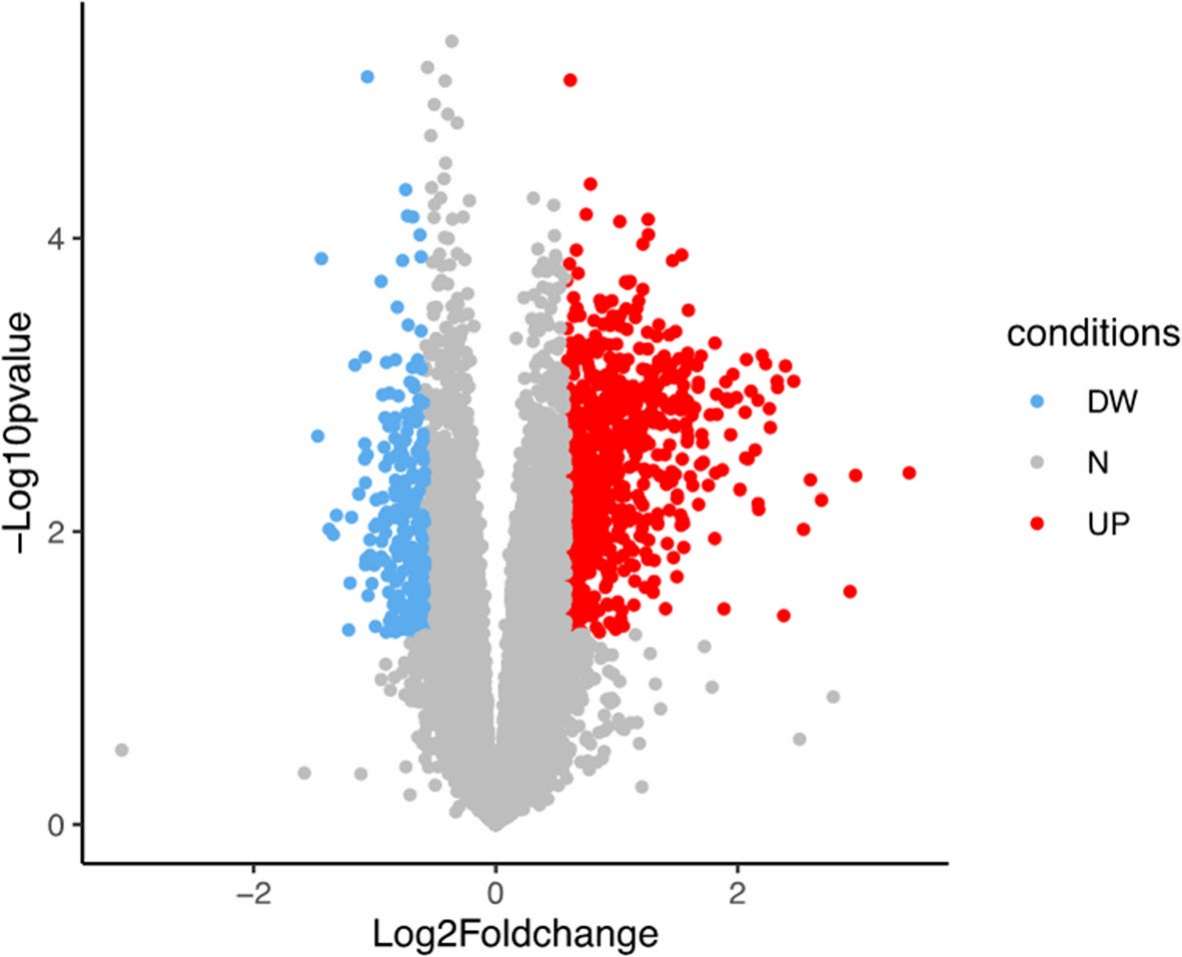
Volcano map represents differentially expressed genes between the ruptured BAVM group and the un-ruptured BAVM group. DW, downregulated; UP, upregulated; N, not changed

A total of 951 genes were differentially expressed between the ruptured and un-ruptured BAVM groups (*P* < 0.05; fold change > 1.5 or < 0.67), of which 740 (77.8%) were upregulated and 211 (22.1%) genes were downregulated in ruptured BAVMs (Fig. 2). The top 5 upregulated and downregulated DEGs (based on the fold changes) were summarized in Table 2. Notably, all of the 5 upregulated DEGs (HMOX1, PLA2G7, FUCA1, ACP5, and IFI30) were related to an inflammatory process or oxidative stress injury. Meanwhile, among the top 5 downregulated genes, TNFSF18 and CCL4L1 belong to cytokines; TPH1 encodes a member of the aromatic amino acid hydroxylase family, which plays crucial roles in the biosynthesis of serotonin [10]; PKHD1L1 could encode a receptor with inducible T lymphocyte expression [11], and TMEM235 encodes a membrane protein which is over-expressed in the brain.

**Table 2:** The top 5 upregulated and downregulated genes between the ruptured BAVM group and the un-ruptured BAVM group

Genes	Up- or downregulation	Fold change	*P*-value
HMOX1	Upregulation	10.700	0.004
PLA2G7	Upregulation	7.869	0.004
FUCA1	Upregulation	7.618	0.026
ACP5	Upregulation	6.471	0.006
IFI30	Upregulation	6.071	0.004
PKHD1L1	Downregulation	0.361	0.002
TNFSF18	Downregulation	0.368	0.000
TMEM235	Downregulation	0.385	0.001
TPH1	Downregulation	0.394	0.010
CCL4L1	Downregulation	0.401	0.008

### Functional analysis of the DEGs

**Fig. 3:**
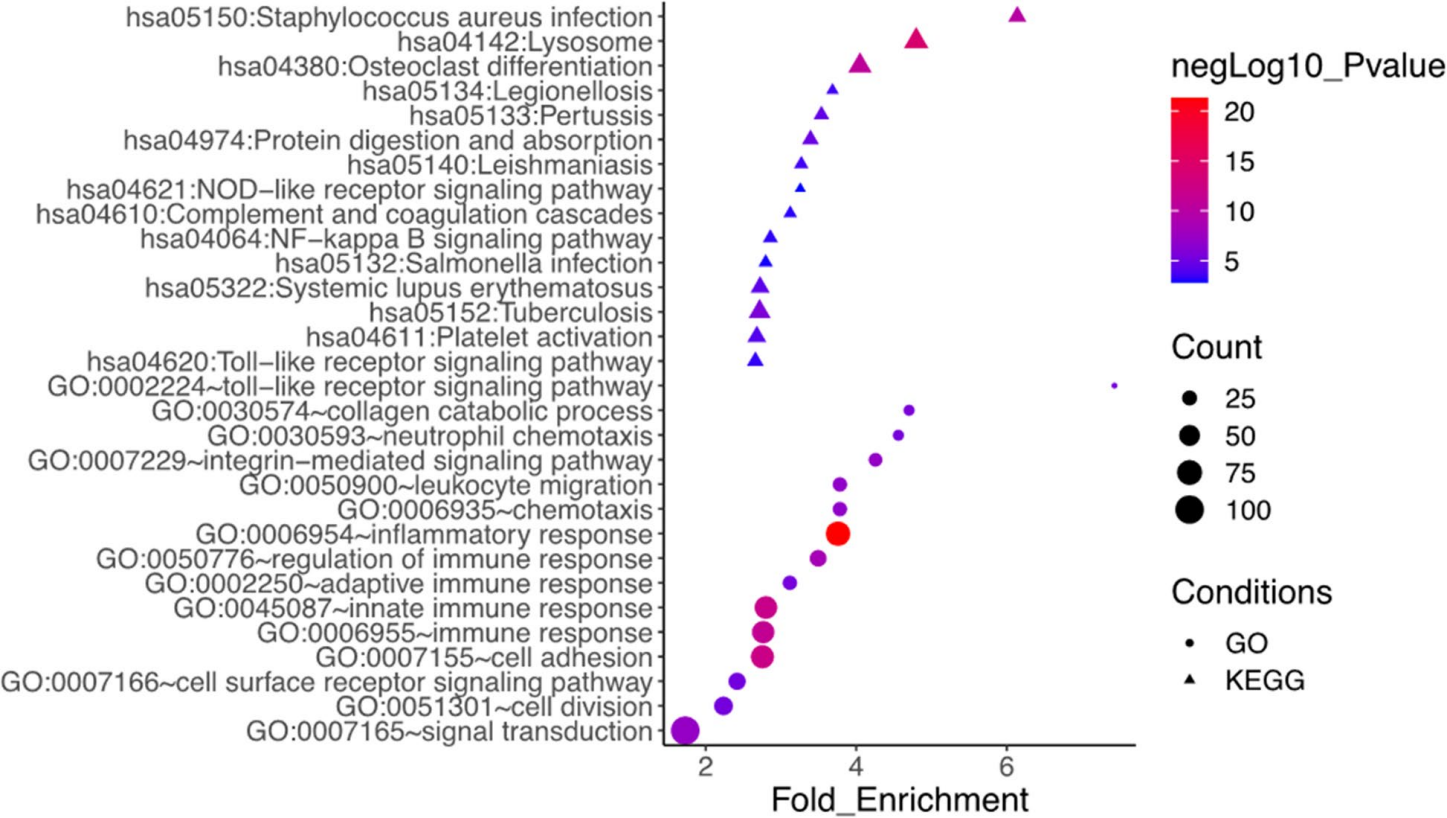
The top 15 GO and KEGG terms upregulated in ruptured BAVMs compared with un-ruptured BAVMs

Functional analysis of the upregulated genes in ruptured BAVM showed the biological processes and pathways related to the inflammatory processes were significantly enriched (Fig. 3, Additional files 2 and 3: Tables S2 and S3). GO analysis demonstrated that several inflammatory terms, including innate immune response and chemotaxis, were highly enriched. In addition, GO terms about extracellular matrix organization were also highly enriched, comprising integrin-mediated signaling pathway and collagen catabolic process, indicating their essential roles in BAVM rupture. KEGG analysis showed inflammation-related diseases and signaling pathways, such as systemic lupus erythematosus, Toll-like receptor signaling pathway, NF-κB signaling pathway, and complement and coagulation cascades which were enriched (FDR-adjusted *P* < 0.05). Meanwhile, some downregulated genes were involved in cell adhesion (DSCAM, CNTN2, CERCAM, ITGBL1, and LAMA3) and genes participating in myofibril assembly (CAPN3, MYOZ1, and MYOZ2). GO and KEGG analysis showed the downregulated genes were also enriched in nervous system development, possibly indicating an important parenchymal impact of BAVM rupture (Additional files 4 and 5: Tables S4 and S5).

### Early rupture-prone DEGs by univariate Cox regression analysis

One hundred twenty-five genes were correlated with the early rupture of BAVMs, among which 96 were upregulated and 29 were downregulated. GO analysis showed that inflammatory response and innate immune response were significantly enriched (Additional file 6: Table S6). There were no KEGG terms from upregulating genes, and GO and KEGG terms from downregulating genes were significantly enriched.

## Discussion

In the ruptured BAVM group, a large cohort of upregulated genes was involved in inflammatory processes, including recruited leukocytes, enhanced cytokines, and leukocyte migration, contributing to vessel wall damage and weakening [12]. Notably, the innate immune responses were highly upregulated in ruptured BAVMs, indicating the crucial role of the innate immune system in BAVMs rupture processes. The cell components of the innate immune system mainly include monocytes, macrophages, and highly phagocytic neutrophils [13], among which macrophage load in BAVM nidus was correlated to higher hemorrhage risk by using ferumoxytol-enhanced MRI [14]. Another study found a higher density of M2 macrophages in the perinidal dilated capillary network (PDCN) in the ruptured BAVM, and it postulated M2 macrophages could lead to an inappropriate BBB which was prone to bleed [15]. Toll-like receptors (TLRs) have crucial roles in activating the innate immune system [16]. As a member of the TLR family, TLR4 is mainly expressed on the cell surface of inflammatory cells, endothelial cells, and smooth muscle cells [17, 18]. TLR4 can bind to MyD88, and the binding between these two proteins leads to downstream expression of pro-inflammatory cytokines [19]. It has been shown that the pathway involving TLR4 and MyD88 promotes tissue remodeling and rupture of a brain aneurysm [20]. In this study, the TLR4 signaling pathway and MyD88-dependent Toll-like receptor signaling pathway were upregulated in ruptured BAVMs. Except for innate immune responses, several other inflammation-related pathways were also increased in the ruptured BAVM group. For instance, NF-κB is a transcription factor that could functionally regulate several pro-inflammatory genes. NF-κB p65, a subunit of NF-κB, was found at a higher level in the ruptured BAVM group compared to the un-ruptured BAVM group [21]. Consistently, in our study, the NF-κB signaling pathway was upregulated in ruptured BAVMs. Several DEGs identified in this study were also tested in the peripheral circulation blood [22]. For instance, TNFAIP6 were found both in ruptured BAVM tissue and peripheral blood. This protein plays a crucial role in the protease network associated with inflammation and is also involved in extracellular matrix (ECM) remodeling, cell adhesion, and cell migration [23]. It is indicated that these DEGs had the potential as biomarkers for the BAVM rupture.

Moreover, extracellular matrix organization was also activated in ruptured BAVMs. The genes involved in this process include COL15A1, COL11A1, COL1A1, COL3A1, COL1A2, COL5A1, COL5A2, COL6A1, and COL6A3, all of which encode subtypes of collagen that constitute the ECM basement membrane and interstitial matrix [24]. Matrix metalloproteinases (MMPs) can degrade all kinds of extracellular matrix proteins and can process a number of bioactive molecules. MMP9 has been reported causing damage to the BAVM vessel walls, leading to rupture of the nidus [25]. In our study, MMP2, instead of MMP9, was increased in ruptured BAVMs, possibly indicating its important role in BAVM rupture. In addition, several genes encoding the activities of the central nervous system, such as MOG, ZBTB16, CNTN2, and MAL, were downregulated in ruptured BAVMs. Due to the abnormal vasculature of the blood vessels in BAVM, the perinidal brain parenchyma, which mainly includes the glial cells, astrocytes, and neurons, is highly affected [26]. We hypothesized that destabilizing BAVM which had increased hemorrhage risk would affect more on the brain parenchyma and result in abnormal gene expressions and cell functions.

## Conclusion

In conclusion, the upregulated genes in the ruptured BAVM group were involved in inflammatory processes and extracellular matrix organization, while some of the downregulated genes participated in cell adhesion and myofibril assembly, indicating the role of enhanced inflammation and reduced vessel strength in BAVMs rupture.

## Supplementary Information

**Additional file 1: Table S1.** comparison of baseline between ruptured and un-ruptured BAVM group.

**Additional file 2: Table S2.** GO terms enriched by the up-regulated genes in ruptured BAVMs.

**Additional file 3: Table S3.** KEGG terms enriched by the up-regulated genes in ruptured BAVMs.

**Additional file 4: Table S4.** GO terms enriched by the down-regulated genes in ruptured BAVMs.

**Additional file 5: Table S5.** KEGG terms enriched by the down-regulated genes in ruptured BAVMs.

**Additional file 6: Table S6.** GO terms enriched by the up-regulated BAVM early rupture-prone genes.

**Additional file 7: Fig. S1.** The typical CT\MRI images of un-ruptured and ruptured BAVM.

## Data Availability

Please contact the authors for data requests.

## Abbreviations

BAVM: Brain arteriovenous malformation
ICH: Intracranial hemorrhage
OS: Overall survival
DEGs: Differential expressed genes
GO: Gene Ontology
KEGG: Kyoto Encyclopedia of Genes and Genomes
ECM: Extracellular matrix
