# Supplementary figures and images for: RNA sequencing analysis between ruptured and un-ruptured brain AVM
Source: Chin Neurosurg J. 2022 Jun 2;8:13. doi: 10.1186/s41016-022-00282-4 (PMC9161579; doi:10.1186/s41016-022-00282-4)

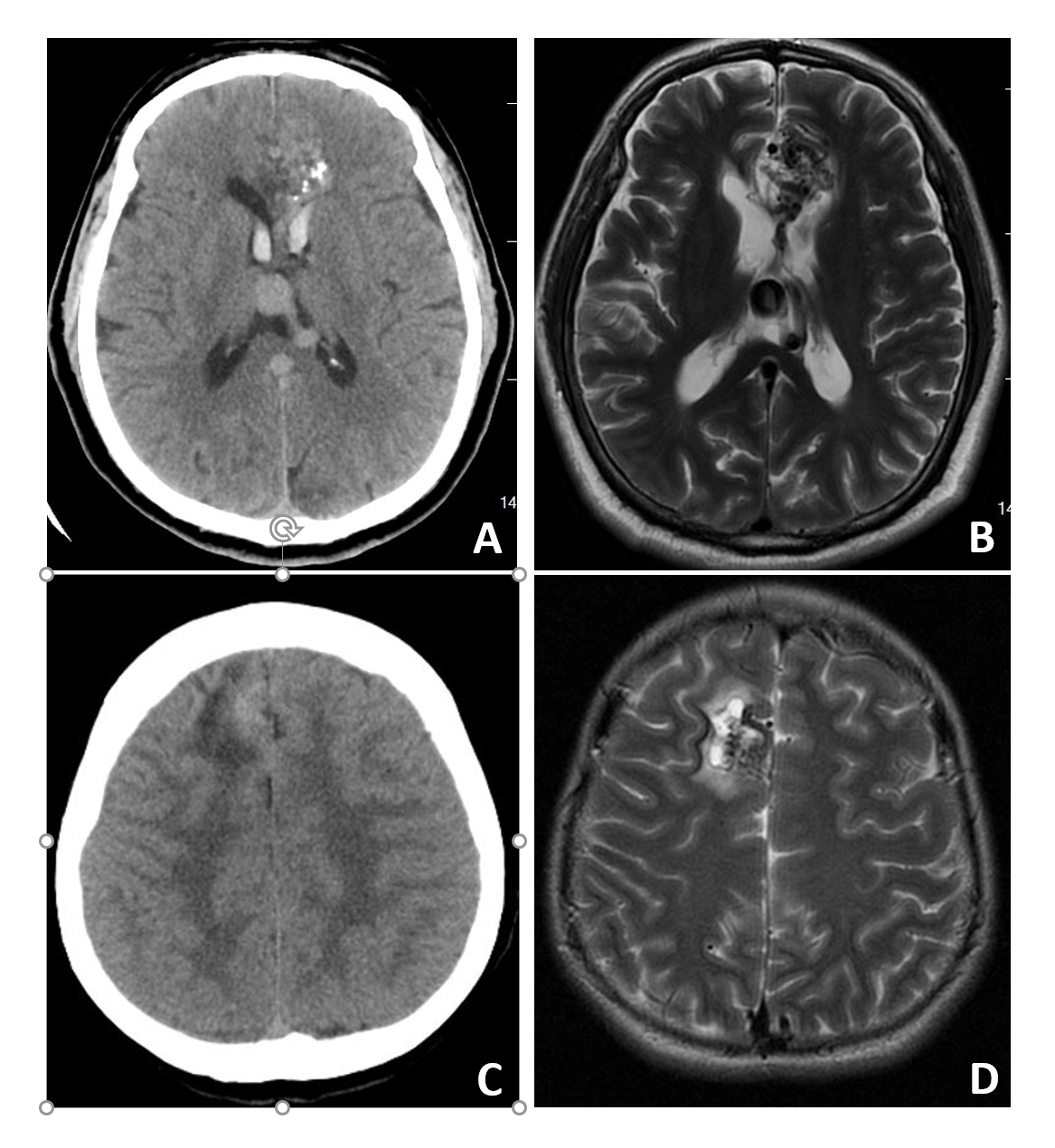

Supplement: Supplementary file 7 — Additional file 7: Fig. S1. The typical CT\MRI images of un-ruptured and ruptured BAVM. [file 41016_2022_282_MOESM7_ESM.png]
